# Genomic and proteomic characterization of two strains of *Shigella flexneri* 2 isolated from infants’ stool samples in Argentina

**DOI:** 10.1186/s12864-022-08711-5

**Published:** 2022-07-08

**Authors:** Mónica F. Torrez Lamberti, Lucrecia C. Terán, Fabián E. Lopez, María de las Mercedes Pescaretti, Mónica A. Delgado

**Affiliations:** 1 Instituto Superior de Investigaciones Biológicas (INSIBIO), CONICET-UNT, and Instituto de Química Biológica “Dr. Bernabé Bloj”, Facultad de Bioquímica, Química y Farmacia, UNT. Chacabuco 461, 5Q7R+96 San Miguel de Tucumán, Argentina; 2 Centro de Referencia Para Lactobacilos (CERELA-CONICET), Chacabuco 145, 5Q9R+3J San Miguel de Tucumán, Argentina; 3 Universidad Nacional de Chilecito (UNdeC), 9 de Julio 22, F5360CKB Chilecito, La Rioja Argentina

**Keywords:** *Shigella*, Clinical isolates, Epidemiology, Genomic, Proteomic, Virulence, Mobilome

## Abstract

**Background:**

*Shigella* specie is a globally important intestinal pathogen disseminated all over the world. In this study we analyzed the genome and the proteomic component of two *Shigella flexneri* 2a clinical isolates, collected from pediatric patients with gastroenteritis of the Northwest region of Argentina (NWA) in two periods of time, with four years of difference. Our goal was to determine putative changes at molecular levels occurred during these four years, that could explain the presence of this *Shigella*`s serovar as the prevalent pathogen in the population under study.

**Results:**

As previously reported, our findings support the idea of *Shigella* has a conserved “core” genome, since comparative studies of CI133 and CI172 genomes performed against 80 genomes obtained from the NCBI database, showed that there is a large number of genes shared among all of them. However, we observed that CI133 and CI172 harbors a small number of strain-specific genes, several of them present in mobile genetic elements, supporting the hypothesis that these isolates were established in the population by horizontal acquisition of genes. These differences were also observed at proteomic level, where it was possible to detect the presence of certain secreted proteins in a culture medium that simulates the host environment.

**Conclusion:**

Great similarities were observed between the CI133 and CI172 strains, confirming the high percentage of genes constituting the “core” genome of *S. flexneri 2*. However, numerous strain specific genes were also determined. The presence of the here identified molecular elements into other strain of our culture collation, is currently used to develop characteristic markers of local pathogens. In addition, the most outstanding result of this study was the first description of a *S. flexneri* 2 producing Colicin E, as one of the characteristics that allows *S. flexneri* 2 to persist in the microbial community. These findings could also contribute to clarify the mechanism and the evolution strategy used by this pathogen to specifically colonize, survive, and cause infection within the NWA population.

**Supplementary Information:**

The online version contains supplementary material available at 10.1186/s12864-022-08711-5.

## Background

*Shigella* is a gram-negative, enteroinvasive and immobile bacillus, established as the etiological agent of the bacillary dysentery commonly known as shigellosis [1, 2]. Shigellosis is an inflammatory bowel syndrome, mainly affecting the colon and caused exclusively by *Shigella*. Usually under infection, the colon undergoes cell modifications causing the water loss as diarrhea, normally with mucus and blood. This disease only occurs in humans and can be transmitted directly by fecal–oral route, from an infected patient to a healthy person, or directly through contaminated food and/or water. Bacillary dysentery is a highly contagious infection and can be acquired with the ingestion of only 100 microorganisms, representing a very low infectious dose [3–7].

The genus *Shigella* (*S*.) involves four species or subgroups, *S. flexneri*, *S. sonnei*, *S. dysenteriae* and *S. boydii* with a wide serotype distribution among them. It has been reported that *S. boydii* covers 20 serotypes, followed by *S. dysenteriae* with 15 serotypes and *S. flexneri* with 19 serotypes [8]. However for *S. sonnei* only one possible serotype has been reported [3]. Worldwide, shigellosis affects annually 164.7 million people, resulting in a high mortality for children younger than 5 years old, especially in low- and middle-income countries [9–11]. In Argentina, it has been reported about 1,000,000 cases of infectious diarrhea per year since 2015 [12, 13]. Many reports provide evidence supporting that *Shigella* species are geographically stratified, distributed according to the level of economic development of a given country [3, 5, 14]. In the western hemisphere, *Shigella* infections were traditionally mostly travel-related, but recent surveillance data indicate a shift to domestically circulating strains [15–17], some of which are increasingly resistant to ciprofloxacin and azithromycin. In addition, different authors have proposed that this pathogen can be used as an index of the hygiene level in a population [7, 18]. However, it is more appropriate to associate *Shigella* infections incidence with the insufficient condition of hygiene and sanitation. Therefore, whereas shigellosis is caused predominantly by *S. flexneri* and *S. dysenteriae* 1 in developing countries, *S. sonnei* is more frequently associated with outbreaks of shigellosis in industrialized countries [7, 18].

The first genome sequence of *S. flexneri* was reported in China by Jin et al. 2002. At present, there is a great number of *S. flexneri* genome sequences reported, currently there are more than 700 sequences uploaded into the data bases of GenBank. Since we previously reported that *S. flexneri* serotype 2 is endemic and the prevalent pathogen in the NWA region [19], here we performed the whole genome sequencing analysis of two *S. flexneri* 2 strains isolated from children suffering diarrhea in Argentina. The goal of this work was to determine the main differences at genomic and proteomic levels, including antibiotic resistance aspects, of *S. flexneri* strains circulating in the NWA region compared with those analyzed in other parts of the world. The acquisition of this knowledge could allow us to identify molecular markers that permit a more rapid and effective pathogen presence detection in patients with gastroenteritis.

## Results

### General features of the genomes and comparison

Between 2013 and 2017, 2,261 *Shigella sp.* strains were isolated in the NWA region, where the prevalent serotype identified was *Shigella flexneri* 2 (*S*. *flexneri* 2) [19]. In that work, we determined that *S. flexneri* serotype 2 displaced serotype AA479 along the last years, highlighting the importance of the emergence of serotype 2 [19]. In order to deeper characterized the prevalent serotype of the region, and to detect molecular markers between them and within isolated strains of other regions of the world, in this work we performed the whole genome sequencing analysis of two *S. flexneri* 2 clinical isolates (CI). The CI133 and CI172 represent the most characteristic phenotypic groups found in the region [19]. The strains were isolated from hospitals belonging to the two main capital cities of the NWA region. The CI133 strain was isolated during the beginning of this study (2013 year); while CI172 was isolated in the last year of study, 4 years later than CI133 (2017 year).

**Fig. 1:**
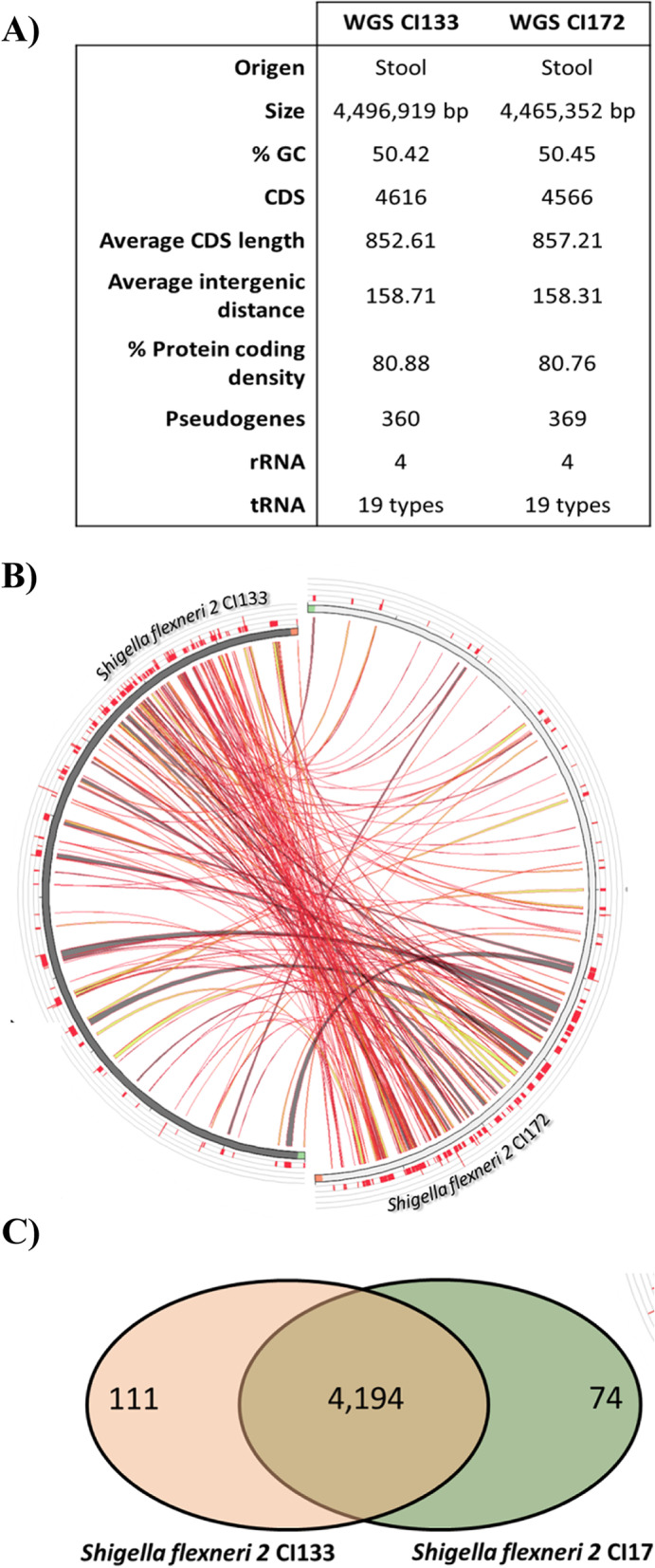
Comparison of the newly sequenced *S. flexneri 2* strains. **A** General features of the *S. flexneri* 2 CI133 and CI172 genomes of sequences. **B** Comparison between CI133 and CI172 strains genome sequences. The strains are represented by the circumference, while the red ribbons represent the alignments of 100% identity performed by BLAST, the width indicate the alignment length. When the alignment is in opposite orientation of the DNA sequence is indicated by twisted lines in grey colors. **C** Venn diagram of *S. flexneri* 2 CI133 and CI172 strains, showing 4,194 shared CDSs at the intersection, the 111 strain specific genes of CI133 represented with an orange circle and the 74 strain specific genes of C172 represented with a green circle

The primary genome features of CI133 and CI172 strains are summarized in Fig. 1A. The whole genome of CI133 strain is composed of a 4,496,919 bp chromosome defined in 350 contigs, while the CI172 genome consist of 4,465,352 bp, included in 336 contigs. There were determined 4,616 coding sequences and 360 pseudogenes in the sequenced genome of CI133 strain, whereas for CI172 strain, 4,566 coding sequences and 369 pseudogenes were identified (Fig. 1A). For both strains, 19 tRNA types were also identified (Fig. 1A). As shown in Fig. 1B (red ribbons), numerous regions with great similarity between both sequences (100% of identity) were also determined. In addition, we observed that CI133 and CI172 strains have in common 4,194 genes displaying 80% identity and 80% coverage, while 112 and 74 were strain specific genes, respectively (Fig. 1C, Table S1). Among these specific genes, in the CI133 strain it is worth noting the identification of the *mdtL* and *yeaN* genes, which are members of the Major Facilitator Superfamily (MFS) (Table S1, in bold). On the other hand, in CI172 genome it is important to highlight the identification as strain-specific genes those encoding for the production and the immunity system of a colicin-like bacteriocin, as well as the toxin-antitoxin (TA) RelEB system (Table S1, in bold).

### Comparative analysis with other *S. flexneri* 2 strains

**Fig. 2:**
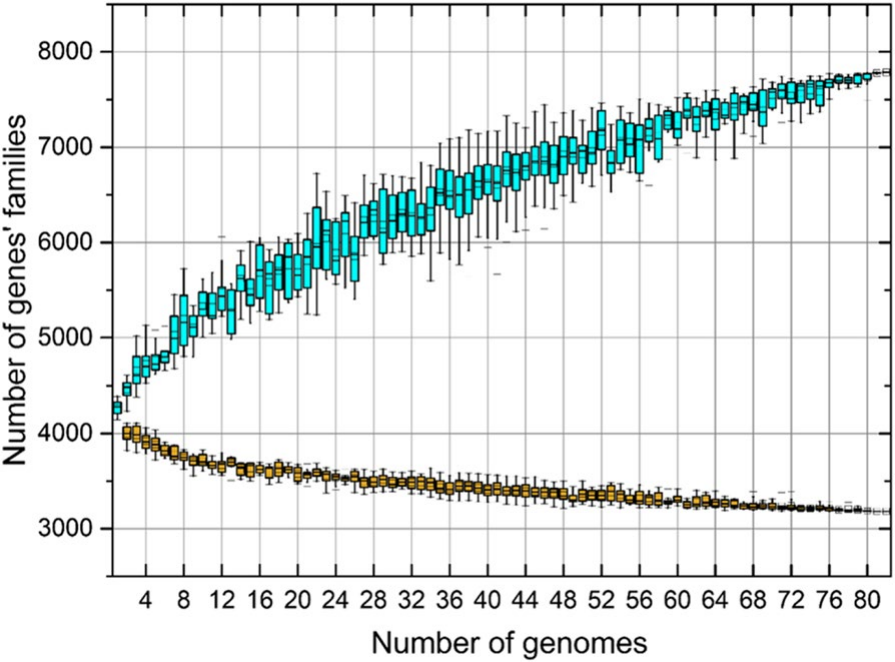
Differential analysis of the CI133 and CI172 genomes sequence. Pangenomic diversity of the newly sequenced strains of *S, flexneri* 2 and 80 genomes of NCBI database. Boxplots represented with light blue represent the increasing pangenomic diversity with the addition of the strains whiles the “core” genome is represented by orange boxplots that decreases with the addition of the strains

A goal of this work was to determine changes in the genome of the prevalent pathogen from the NWA region to elucidate the main virulence factors that allow us the development of molecular strategies for its eradication, as mentioned earlier. To do this, we analyzed 80 *S. flexneri* 2 genomes deposited in the GenBank database from NCBI, which were compared with the genome of the two isolates obtained and sequenced in this work. As shown in Fig. 2, the pangenome of *S. flexneri* 2’ consists in 7,789 families of genes (in blue). From that whole set of genes, 3,182 genes families, represent the “core” genome (in yellow), while the last 4,607 families of genes represent the variable genome (41% and 59% of the genome, respectively). These results show conservation among the genomes of the 82 strains of *S. flexneri* 2 studied (41%). The number of strain specific genes go from no genes in strains 4028STDY6330022, 4028STDY6330041, 2012ZH074 and 2012ZH118 of *S. flexneri* 2 to 293 genes in *S. flexneri* 2 strain 1508 (Table S2, CI133 and CI172 are highlighted in bold). In this analysis, we observed that the CI133 strain have 11 strain-specific genes, while in CI172 we identified 18 strain-specific genes. In addition, these specific genes codify for unknown function proteins (Table 1). Interestingly, among the 80 genomes studied we found that the CI172 strain has as unique genes the cluster encoding for Colicin E, consisting in the production and immunity to the bacteriocin, as well as the lytic protein (Table 1). Moreover, when a phylogenetic analysis of 15 molecular markers was carried out as described in Material and Methods, we observed that the CI172 strain was located in a separate branch, while the other remaining strains were group together as a sister group in which is included the *S. flexneri* 2a ATCC 29,903 type strain (Fig. S1). Particularly, the CI133 strain was clustered with 4028STDY6330022 and 4028STDY6330016, both isolated in France in 2010 (Fig. S1).

**Table 1:** Strain-specific genes of CI133 and CI172 strains

**Type**^a^	**Gene name**	**Begin**	**End**	**Frame**	**Product**
**CI133 strain**					
CDS	_	2,465,413	2,465,574	1	conserved protein of unknown function
CDS	_	3,383,063	3,383,389	-3	Coat protein (fragment)
fCDS	*yjcF*	3,455,090	3,455,269	-3	fragment of conserved hypothetical protein (part 2)
CDS	_	3,529,696	3,529,758	1	protein of unknown function
fCDS	*yidX*	872,107	872,283	1	fragment of putative lipoprotein C (part 2)
CDS	_	4,151,583	4,151,867	3	conserved protein of unknown function
CDS	_	4,157,162	4,157,227	2	protein of unknown function
CDS	_	1,847,378	1,847,581	2	protein of unknown function
CDS	_	305,872	305,958	-1	conserved protein of unknown function
fCDS	*ycgV*	312,055	312,423	1	fragment of putative adhesin; putative autotransporter (part 1)
fCDS	*rcsD*	2,721,543	2,722,163	-1	fragment of phosphotransfer intermediate protein in two-component regulatory system RcsCDB (part 1)
**CI172 strain**					
CDS	_	382,222	382,428	-3	Conserved protein of unknown function
CDS	_	384,019	386,238	1	Conjugal transfer protein
CDS	*ceaB*	386,471	388,216	2	Colicin-E2
CDS	*ceiB*	388,219	388,479	1	Colicin-E2 immunity protein
CDS	*celB*	388,541	388,684	2	Lysis protein for colicins E2 and E3
CDS	_	388,954	389,022	1	Protein of unknown function
fCDS	*mtlA*	2,929,888	2,930,892	-3	Fragment of fused mannitol-specific PTS enzymes: IIA components; IIB components; IIC components (part 1)
CDS	_	3,064,292	3,064,357	2	Protein of unknown function
CDS	_	3,239,766	3,239,861	-1	Conserved protein of unknown function
CDS	_	496,366	496,518	1	Protein of unknown function
CDS	_	556,204	556,617	-3	Protein of unknown function
fCDS	*ygbI*	3,578,405	3,578,527	-2	Fragment of putative DNA-binding transcriptional regulator (part 1)
CDS	_	3,671,334	3,671,399	3	Conserved protein of unknown function
CDS	_	3,883,260	3,883,328	3	Conserved protein of unknown function
fCDS	*yjjI*	946,318	946,470	1	Fragment of conserved hypothetical protein (part 2)
CDS	_	1,052,082	1,052,255	-1	Protein of unknown function
CDS	_	1,535,495	1,536,028	-2	Conserved protein of unknown function
fCDS	*pdxK*	2,235,680	2,235,982	2	Fragment of pyridoxal-pyridoxamine kinase/hydroxymethylpyrimidine kinase (part 1)

^a^*CDS* Coding DNA sequence, *fCDS* Framshifted coding DNA sequence

### Antimicrobial susceptibility profiles and antibiotic resistance genes identified in both strains

Using the Comprehensive Antibiotic Resistance Database (CARD) we analyzed the presence of genetic antibiotic resistance determinants, which could be used as differential markers. We observed that both strains shared similar profile of antibiotic resistance genes. This analysis allowed us to determine six antibiotic resistance determinants in the sequence of the CI133 strain genome, while in CI172 strain five genes codify for antibiotic resistance. As shown in Table 2A, a perfect match for *cat*, *evgA*, *sat*, *tetD* and *hns* resistance genes in both genomes were determined. These genes codify for the resistance to chloramphenicol (*cat*, phenicol), norfloxacin (*evgA*, fluoroquinolone), antibiotics targeting nucleosides (*sat*, such as quinolones), tetracycline (*tetD*), and carbapenems in general (*hns*) [20]. However, *marA* gene was identified only in CI133 genome, pointing out a difference between both strains. In addition, the sequences were analyzed using second software, ResFinder, which allows the identification of functional metagenomic antibiotic resistance determinants. This analysis confirmed the profile and location of the drug resistance genes in CI133 strain (Table 2B). While in the CI172 genome the chloramphenicol resistance gene could not be detected using this tool (Table 2B). To investigate if those genes awarded a resistant phenotype, we performed antibiotic susceptibility tests using antibiotic discs. A set of 10 antibiotics was tested, including the ones which resistance genes were identified in both genomes. As shown in Table 3, both strains shared a similar pattern of antibiotic resistance, showing “in vitro” resistance to chloramphenicol, ampicillin, tetracycline, streptomycin, vancomycin, and imipenem. In addition, CI172 was also resistant to trimethoprim/sulfamethoxazole while CI133 was susceptible, even though the *dfrA1* gene which encodes resistance to trimethoprim/sulfamethoxazole was identified by ResFinder in this strain. Therefore, we confirmed “in vitro” the functionality of the genes identified in both genomes by bioinformatics tools.

**Table 2:** Antibiotic resistance genes identified in CI133 and CI172 strains

**A-CARD softwad results**					**CI133 strain**			**CI172 strain**		
**Gene**	**Product**	**Resistance**	**Hit Type**	**Ident (%)**	**Score**	**E-value**	Contig*	**Score**	**E-value**	Contig*
*cat*	Chloramphenicol acetyltransferase	ARO:3,000,387: phenicol antibiotic	Perfect	100	465,692	4,60E-162	NODE_154	465,692	5,00E-165	NODE_208
*hns*	Global nucleic acid-binding transcriptional dual regulator H-NS	ARO:3,000,008: penam	Perfect	100	276,944	2,56E-88	NODE_17	276,944	3,00E-92	NODE_462
*evgA*	DNA-binding response regulator in two-component regulatory system EvgAS	ARO:3,000,662: norfloxacin	Perfect	100	417,157	1,93E-142	NODE_106	417,157	2,00E-145	NODE_158
*tetD*	Transposon Tn10 TetD protein	ARO:0,000,051: tetracycline	Perfect	100	283,493	7,89E-92	NODE_624	283,493	8,00E-95	NODE_307
*sat*	Streptothricin acetyltransferase	ARO:3,000,034: nucleoside antibiotic	Perfect	100	365,925	2,88E-123	NODE_170	365,925	3,00E-126	NODE_6
*marA*	DNA-binding transcriptional dual activator of multiple antibiotic resistance	ARO:0,000,001: fluoroquinolone antibiotic	Perfect	100	266,544	1,63E-84	NODE_122	**ND**	**–––**	**–––**
**B- ResFinder:** Identifcation of acquired antibiotic					**CI133 strain**			**CI172 strain**		
**Gene**	**Phenotype**		**Accession N**^**o**^		**Identity**	**Contig**	**Position in contig**	**Identity**	**Contig**	**Position in contig**
*blaOXA-1*	Amoxicillin, amoxicillin + clavulanic acid, ampicillin, ampicillin + clavulanic acid, cefepime, piperacillin, piperacillin + tazobactam		HQ170510		100	NODE_154	7038..7868	100	NODE_208	113..943
*catA1*	Chloramphenicol		V00622		100	NODE_154	303..962	**ND**	––	––
*aadA1*	Spectinomycin, streptomycin		JQ480156		100	NODE_170	1950..2738	100	NODE_6	3482..4270
*dfrA1*	Trimethoprim		X00926		100	NODE_170	3415..3888	100	NODE_6	2332..2805
*mdf(A)*	Unknown macrolide, aminoglycoside, tetracycline, fluoroquinolone phenicol, and rifamycin		Y08743		100	NODE_175	32,621..33847	**ND**	––	––
*sitABCD*	Hydrogen peroxide		AY598030		100	NODE_224	596..4045	**ND**	––	––
*tetB*	Doxycycline, tetracycline, minocycline		AF326777		100	NODE_624	1153..2358	100	NODE_307	1151..2356

*ND* Not determined, Contig* The contig containing drugs determinant resistance gene was identified by BlastN

**Table 3:** Phenotype of antibiotic resistance profiles displayed by CII33 and CI172 strains

ANTIBIOTIC	CI133	CI172
CHLORAMPHENICOL	R	R
AMPICILLIN	R	R
TRIMETHOPRIM/SULFAMETHOXAZOLE	S	R
FOSFOMYCIN	S	S
FURAZOLIDONE	S	S
TETRACYCLINE	R	R
KANAMYCIN	S	S
CIPROFLOXACIN	S	S
STREPTOMYCIN	R	R
VANCOMYCIN	R	R
NALIDIXIC ACID	S	S
IMIPENEM	R	R
GENTAMICIN	S	S

*R* Resistant, *S* Sensitive

### Pathogenesis and virulence factors

*Shigella* pathogenesis and virulence were previously well described [21, 22]. It is known that *Shigella* pathogenicity depends on different chromosomal loci as well as on a mega virulence plasmid harboring the main virulence factors [23, 24]. To investigate the presence of such virulence determinants and the possible differences between both sequences, we used VirulenceFinder database to perform a BLAST screening of the assembled genomes of CI133 and CI172 strains. As a result of this analysis several virulence factors were identified. As shown in Table 4, 10 virulence genes, located in different contigs, were identified in CI133 and 11 in CI172, all of them with 99–100% of identity. The virulence factors identified in both genomes include the transcriptional activator VirF (*virF*), *Shigella* IgA-like protease homologue (*sigA*), *Shigella* extracellular protein A (*sepA*), serine protease autotransporters of *Enterobacteriaceae* (*pic*), long polar fimbriae (*lpfA*), invasion plasmid antigen (*ipaH9.8*), invasion protein (*ipaD*), glutamate decarboxylase (*gad*), hexosyltransferase homolog (*capU*A), and the iron transport protein (*sitA*). In addition, the presence of *celB* gene as virulence factor was only determined in the genome of CI172 strain. This last gene codifies for the Endonuclease colicin E2, which is in concordance with the identification of genes encoding for the production and the immunity system to colicin E2 among the strain-specific genes of CI172 (Table 1). The presence of these genes supports and proves the pathogenic phenotype of these strains, which were isolated from patients suffering diarrhea.

**Table 4:** Virulence genes identified in the CI133 and CI172 strains

**VirulenceFinder**			**CI133 strain**			**CI172 strain**		
**Virulence factor**	**Protein function**	**Accession Nº**	**Contig**	**Position in contig**	**Identity (%)**	**Contig**	**Position in contig**	**Identity (%)**
*virF*	VirF transcriptional activator	AF348706	NODE_249	1098..1907	99.88	NODE_150	80..889	100
*sigA*	*Shigella* IgA-like protease homologue	AE005674	NODE_458	8272..12129	99.97	NODE_263	8265..12122	100
*sepA*	*Shigella* extracellular protein A	CP001384	NODE_73	362..4456	100	NODE_1	1304..5398	100
*pic*	Serine protease autotransporters of *Enterobacteriaceae* (SPATE)	CP003289	NODE_458	706..4824	100	NODE_263	704..4822	100
*lpfA*	Long polar fimbriae	AE014073	NODE_477	357..929	100	NODE_434	829..1401	100
*ipaH9.8*	Invasion plasmid antigen *Shigella flexneri*	CP0226	NODE_199	2485..3222	99.86	NODE_1201	1..831	100
*ipaD*	Invasion protein *Shigella flexneri*	CP001384	NODE_103	6422..7420	100	NODE_21	26,156..27154	100
*gad*	Glutamate decarboxylase	AE005674/ CP000266	NODE_453	9318..10718	100	NODE_305/ 372	6210..6771/ 9316..9877	100/ 100
*capU*	Hexosyltransferase homolog	CP001062	NODE_132	2175..3263	99.72	NODE_65	4347..5435	99
*sitA*	Iron transport protein	UFYN01000008	NODE_224	3131..4045	100	NODE_1118	3747..4661	100
*celb*	Endonuclease colicin E2	D00021	ND	ND	ND	NODE_16	6370..6513	100

*ND* Not determined

### Mobile elements

#### Plasmids

There are several reports describing the plasmids identified by sequencing in different *Shigella sp* strains [25–28]. These elements constitute one of the main sources of genetic variation between different bacterial species and within same species. The identification of these plasmids represents an important tool for the construction of molecular markers. An “in silico” search for plasmids was carried out using the PlasmidFinder platform. Through this tool we identified the presence of several contigs related with plasmids in these strains. As shown in Table 5, on both genomes the IncFII plasmidic incompatibility element was detected, suggesting that both strains may harbor the same plasmid. In addition, on CI172 sequence the Col156 and ColRNAI elements were also identified within the contigs 16 and 40, respectively. These findings further highlight the difference between both strains.

**Table 5:** Plasmidic elements identified in the CI133 and CI172 strains

		**PlasmidFinder**	**Organism: *Enterobacteriaceae***		
**Plasmid**	**Identity**	**Query / Template length**	**Contig**	**Position in contig**	**Accession Nº**
			**CI133 strain**		
IncFII	96.17	261 / 261	NODE_263_length_3428_cov_137.611145	1720..1979	AY458016
			**CI172 strain**		
Col156	96.05	152 / 154	NODE_16_length_6768_cov_3394.772949	750..901	NC009781
IncFII	96.17	261 / 261	NODE_259_length_3426_cov_250.150024	1528..1787	AY458016
ColRNAI	93.18	87 / 90	NODE_40_length_4009_cov_2266.553955	9..96	DQ298019

**Fig. 3:**
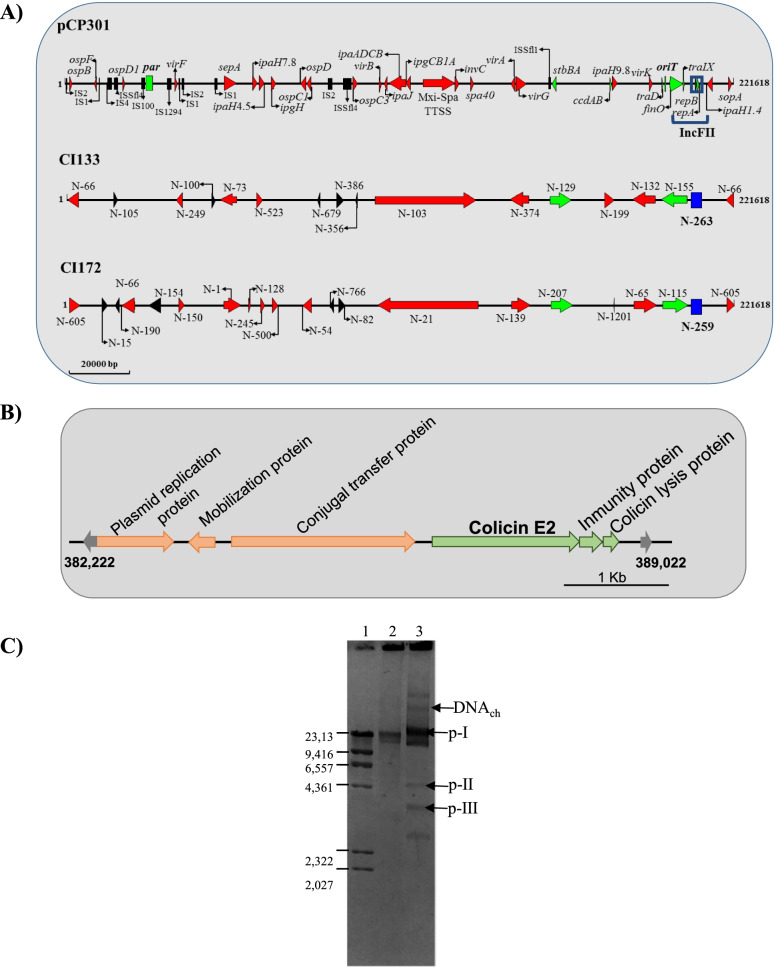
Plasmidic content analysis from genome sequence of the CI133 and CI172 strains. **A** Scale reconstruction of the virulence plasmid identified in the CI133 and CI172 strains, by homology of the sequences obtained with pCP301 and using the DNAMAN program. Red arrows indicate virulence genes identified in each strain; Green arrows indicate elements required for plasmid stability, black arrows indicate insertion sequences and the blue box indicates the origin of replication. The direction of the arrows indicates the orientation of each element with respect to the pCP301 sequence. The node or contigs where each element is found in each strain is indicated on each element. **B** Strain specific cluster of *S. flexneri* 2 CI172 strain showing the Colicin related genes and their probable plasmidic origin. **C** DNA plasmidic profile obtained from virulent CI133 and CI172 strains: 1-λDNA-HindIII molecular marker used to estimate the size of each plasmidic band, the size of each band is in the left indicated; 2- CI133 strain and 3- CI172 strain. The analysis was carried out by agarose gel electrophoresis and stained with ethidium bromide. DNA_ch_: chromosomal DNA; p-I, pII and p-III putative plasmids identified by PlasmidFinder software

In order to test if IncFII belongs to the virulence plasmid, the search was extended through contig 263 (CI133) and contig 259 (CI172) alignment with BLASTn, using the complete plasmid databases of NCBI. In both alignments 100% of identity with a specific region of pCP301 plasmid (GenBank accession number NC_004851.1) was obtained. The pCP301 was described by Jin, Q et al*.* (2002) as a 221,618 bp virulence plasmid of *S*. *flexneri* 2a [29]. To uncover the location of the plasmidic loci that potentially contribute to the virulence of the under-study strains, we performed a new alignment using the pCP301 complete sequence and those obtained for the CI133 and CI172 strains. These results allowed us to reconstitute the putative pCP301 plasmid harbored in CI133 and CI172. As shown in Fig. 3A, 16 contigs of the CI133 strain sequence showed homology with different regions of the pCP301 plasmid sequence (99–100% identity), including the contig 263 which harbors the IncFII element (blue box). We also observed that 8 of these 16 contigs shared homology with virulence factors, 5 of which were also identified with VirulenceFinder software (Fig. 3A red arrows, Table 4). In addition, 3 contigs displayed homology to pCP301 regions related to the stability, replication and incompatibility functions, and the last 5 contigs with different insertion sequences located on this plasmid (Fig. 3A, green arrows, blue box and black box, respectively). In the same way, the alignment of pCP301 with the CI172 contigs showed a similar profile to CI133, since 21 contigs were involved, including the node 259 harboring the IncFII element (Fig. 3A, blue box). We observed that in CI172 strain, 13 contigs showed homology with regions of pCP301 containing virulence genes (Fig. 3A red arrows). Here, it is important to mention that these virulence gene regions involved 5 of those 13 contigs (Table 4). The remaining 3 contigs presented homologous sequences with regions inherent to plasmid maintenance and replication, and 5 contigs with insertion sequences (Fig. 3A, green arrows and black box, respectively). Together, these results provide evidence of pCP301 presence as the main plasmid that determines the CI133 and CI172 strains’ virulence.

On the other hand, we analyzed the CI172’s contig 16 and 40 in order to characterize those other putative plasmids identified by PlasmidFinder platform. As showed in Table 5, the contig 16 displayed a 96% identity with Col156, and coincidentally in this contig the presence of those genes encoding the colicin E2 system was evidenced. (Table 4). As with virulence genes, BLASTn alignment was performed using the node 16 sequence to identify the presence of a plasmid. The results showed homology with ColE2-P9 plasmid. As was previously reported, ColE2-P9 is a plasmid of about 7 kb identified in about 10 to 15 copies per *Shigella* chromosome [30–33], encoding for the colicin E2 determinant genes. The alignment of pColE2-P9 and Node 16 allowed us to determine that, in this node, the colicin neighbors’ genes displayed homology with plasmid related genes encoding for replication protein and those mobilization/conjugation transfer proteins (MobA/MobL family), suggesting the plasmidic origin of the bacteriocin system (Fig. 3B).

Then, to follow up with the plasmid discovering, we analyzed the contig 40 of the CI172 strain. In addition to the ColRNAI sequence, BLASTn showed 100% identity between the contig 40 sequence and the pSF301-3 plasmid (NC_019250.1). pSF301-3 is a 4,043 kb plasmid isolated from a *S. flexneri* 2a strain, whose replication is regulated by the Rop protein, which acts through modulation of primer RNA precursor transcription [34–36]. Like pColE2-P9, pSF301-3 harbors genes encoding for a mobilization family of proteins such as MobC, MbeB and MbeD, necessary for the specific transference when other conjugative plasmid it is also present into the host strain [37]. The identity observed with this plasmid suggests that the contig 40 is a small plasmid also present into CI172, representing another difference with CI133.

Finally, to experimentally confirm the presence of the mentioned plasmids in the CI133 and CI172 strains we performed the procedure described by Kado and Liu (1981) for identification and isolation of different size plasmids including large and small elements [38]. As shown in Fig. 3C, when the plasmid content of CI133 and CI172 was analyzed and compared against the λDNA-HindIII molecular marker, we were able to infer the number and size of the putative plasmids identified “in silico” in both strains. In agreement with the bioinformatics results, the CI133 strain presented only one band, possibly corresponding to the virulence plasmid of around 22,16 kb (Fig. 3C, arrow). While in the CI172 strain the presence of four bands was observed, three of them could correspond to the 3 identified plasmids, the virulence plasmid (30 kb), the pColE (4 kb) and the pSF301-3 (4 kb) (Fig. 3 C, pI, p-II and p-III, respectively).

#### Prophages

The presence of prophages within the chromosome can allow some bacteria to develop resistance to antibiotics, adapt to new environmental niches, even improve adherence to host and become pathogens. Using the PHAST tool [39], a search for phages in the genomes of CI133 and CI172 strains was carried out. The results showed that in CI133 genome, 4 prophage regions are present as intact elements (Table 6), while other 7 regions are incomplete and 2 regions as questionable elements, making a total of 13 prophage regions identified (Table S3). Furthermore, in the 4 intact prophage regions the left and right *att* phage binding sites to the bacterial chromosome were detected, as well as those genes encoding for the integrase, tail, head, capsid, portal and the terminase (Table S4). The location of these regions in the CI133 obtained sequences, allowed to determine that all the contigs involved in the phage regions are continuously overlapped within each other in the genome (Table 6). In the case of CI172, there were found a total of 12 phage regions, 2 of which are questionable, 7 correspond to incomplete prophages (Table S3) and 3 correspond to intact prophages. In these last 3 regions, all the elements that define a phage, contained in different continuous contigs, were also detected (Table 6, Tables S3 and S4).

**Table 6:** Intact prophage regions identified by PHAST software into the CI133 and CI172 genomes

Region	Region Length	SCORE	CDS N°	Region Position	Contigs involved	Possible Phage	GC (%)
**CI133 strain**							
1	14.5 Kb	100	23	1,273,949–1,288,451	NODE_59_length_95536^a^	PHAGE_Salmon_118970_sal3_NC_031940	50.67%
4	42.9 Kb	150	22	3,351,842–3,394,802	NODE_298_length_13180 NODE_300_length_4451 NODE_303_length_8775 NODE_304_length_4965 NODE_305_length_11191	PHAGE_Phage_Gifsy_1_NC_010392	50.40%
6	38.8 Kb	140	29	3,715,134–3,753,956	NODE_378_length_11128^a^ NODE_379_length_11536 NODE_387_length_2174 NODE_388_length_2179 NODE_389_length_2269 NODE_390_length_3407 NODE_391_length_1539 NODE_394_length_10168 NODE_398_length_21406^a^	PHAGE_Entero_mEp460_NC_019716	50.77%
12	36.4 Kb	100	30	4,298,371–4,334,788	NODE_916_length_55721^a^ NODE_925_length_549 NODE_937_length_1867 NODE_948_length_5860 NODE_950_length_762 NODE_954_length_3661 NODE_976_length_528 NODE_980_length_575 NODE_1017_length_47747^a^	PHAGE_Entero_BP_4795_NC_004813	48.57%
**CI172 strain**							
1	14 Kb	130	21	1,034,005–1,048,079	NODE_60_length_29546^a^ NODE_63_length_8773	PHAGE_Phage_Gifsy_1_NC_010392	51.61%
2	14.5 Kb	100	22	1,119,930–1,134,432	NODE_64_length_94539^a^	PHAGE_Salmon_118970_sal3_NC_031940	50.67%
4	38.3 Kb	110	11	3,001,801–3,040,120	NODE_215_length_64273^a^ NODE_217_length_11752 NODE_218_length_11762 NODE_220_length_1372 NODE_221_length_5430^a^	PHAGE_Entero_mEp460_NC_019716	52.05%

^a^Partial contig sequence involved

In order to confirm the above result, we also analyzed the presence of prophage regions in both strains using PHASTER tool (PHAge Search Tool Enhanced Release). As shown in Table 7, using this new server it was possible to detect 4 prophage regions into the CI133 genome, but just only the region 4 harbor an intact element, while other 1 region is incomplete and 2 regions more represent questionable elements. It is important to mention that the intact prophage identified into the region 4, involving the node 458 sequence (Table 7), was not detected by PHAST, confirming the efficiency of update performed on PHASTER server. Furthermore, in the node 458 sequence all the elements required for a functional phage like the *attL* and *attR* phage binding sites, the integrase, tail, head, capsid, portal and the terminase encoding genes, were detected (Table S4B). Unlike PHAST, the results obtained from PHASTER did not show the presence of intact prophage regions into the CI172 genome, rather than 2 of 4 regions were questionable prophages and 2 more corresponded to incomplete prophages (Table 7).

**Table 7:** Prophage regions identified by PHASTER software into the CI133 and CI172 genomes

**Region**	**Region Length**	**Score**	**CDSs** N°	**Region Position**	**Contigs involved**	**Completeness**	**Most Common Phage**	**GC (%)**
**CI133**								
1	16.7 Kb	90	27	72,788–89,522	NODE_59_length_95536	questionable	PHAGE_Salmon_118970_sal3_ NC_031940	50.53%
2	8.2 Kb	40	11	145–8354	NODE_353_length_8361	incomplete	PHAGE_Shigel_SfII_NC_021857	39.56%
3	19.5 Kb	70	14	1847–21,356	NODE_398_length_21406	questionable	PHAGE_Escher_500465_1_ NC_049342	48.74%
**4**	**23.4 Kb**	**100**	**18**	**155–23,621**	**NODE_458_length_50419**	**intact**	**PHAGE_Entero_BP_4795_ NC_004813**	**47.50%**
**CI172**								
1	10.9 Kb	60	12	223–11,190	NODE_48_length_20601	incomplete	PHAGE_Escher_500465_1_ NC_049342	50.43%
2	16.7 Kb	90	27	71,791–88,525	NODE_64_length_94539	questionable	PHAGE_Salmon_118970_sal3_ NC_031940	50.53%
3	8.2 Kb	40	11	143–8352	NODE_165_length_8359	incomplete	PHAGE_Shigel_SfII_NC_021857	39.56%
4	23.3 Kb	90	16	153–23,485	NODE_263_length_50444	questionable	PHAGE_Entero_BP_4795_ NC_004813	47.73%

Interestingly, when we compared the results of both servers, we observed that in both genomes, the regions of prophages that were questionable with PHASTER involved nodes that were forming part of complete phages identified with PHAST (Table 6 vs Table 7). This led us to hypothesize that these differences were observed since the sequences that we used for the analysis are separated in nodes or contigs and not assembled. To confirm this assumption, the sequence of each node involving one complete phage by PHAST detected, were assembled and uploaded into the PHASTER server. This time, PHASTER identified the same complete phage identified by PHAST. These results indicate that despite of differences between both servers, the results obtained by each are complementary. Therefore, we demonstrate that the AC133 strain has a total of five regions that correspond to five possible complete prophages, while the AC172 strain contains only three.

#### CRISPR systems

CRISPR (Clustered Regularly Interspaced Short Palindromic Repeats) constitute a particular family of repeated sequences in tandem found in a wide range of prokaryotic genomes. The CRISPR consist of a nucleotide sequences located within highly conserved regions that vary in size, are separated by unique sequences of similar size (spacer) and they have generally a viral origin [40]. The systems called CRISPR-Cas (CRISPR-associated Cas proteins) provide the bacteria immunity against invasive genetic elements, such as plasmids and phages, and provides a form of acquired immunity. Therefore, using the online tool CRISPR Finder was possible to search for these CRISPR systems in the sequenced genome of both strains under study. As shown in Table 8, within the CI133 genome 4 questionable CRISPR systems were identified into different contigs, while in CI172 genome 3 of these questionable elements were found. Moreover, when CRISPR-Cas Meta Finder server was applied to the genomes analysis we identified one more of these systems in CI133 and CI172 strains. These elements were located into the Node 125 and 47, respectively (Table 8). Unlike those CRISPR above described these new systems could be functional, since not only displayed sequence homology with the CRISPR Class I family, but also contains the *cas*3_1_I genes and the DR consensus (Table 8). In addition, we observed that the Class I-CRISPR is apparently the same in both strains due to presence of equal DR length and number of spacers. It is important to mention that a more in-depth analysis of these CRISPR immune systems is being carried out in our laboratory.

**Table 8:** CRISPR/Cas systems identified into the CI133 and CI172 genomes

Elements	CRISPR Id/ Cas Type	Contig	CRISPR length	Description
**CI133 strain**				
CRISPR		NODE 94 length_67066	109	CRISPR start position: 22,419 ––––– CRISPR end position: 22,528
				DR consensus: CCGGATAAGCAAAGCGCATCCGGCA
				DR length: 25 Number of spacers: 1
CRISPR		NODE 128 length_34984	117	CRISPR start position: 31,705 ––––– CRISPR end position: 31,822
				DR consensus: CCGAGCCGTAGGCCGGATAAGGCGTTCACGC
				DR length: 31 Number of spacers: 1
CRISPR		NODE 277 length_35192	101	CRISPR start position: 26,959 ––––– CRISPR end position: 27,060
				DR consensus: TTGTTGATGTTGTTGTGTTTTGTA
				DR length: 24 Number of spacers: 1
CRISPR		NODE 754 length_38331	123	CRISPR start position: 8501 ––––– CRISPR end position: 8624
				DR consensus: CGACCCCCACCATGTCAAGGTGGTGCTCTAACCAACTGAGCTA
				DR length: 43 Number of spacers: 1
CRISPR	crispr_1	NODE 125 length_29975	146	CRISPR start position: 29,881 ––––– CRISPR end position: 30,027 DR consensus: TTTGAGGTGTACTGGCAATAGCGGACACTACCATTTGTTCTTTTTTTAAGCAG DR length: 53 Number of spacers: 1
Cas cluster	General-Class1			Start 11,926 End 13,077; Gene name Cas3_1_I, Orientation ( +)
**CI172 strain**				
CRISPR		NODE 3 length_34982	126	CRISPR start position: 3272 ––––– CRISPR end position: 3398
				DR consensus: TTTGTAGGCCTGATAAGACGCGCCAGCGTCGCATCAGGC
				DR length: 39 Number of spacers: 1
CRISPR		NODE 26 length_35304	102	CRISPR start position: 8262 ––––– CRISPR end position: 8364
				DR consensus: TGCGCCAGCATCGCATCCGGCATCA
				DR length: 25 Number of spacers: 1
CRISPR		NODE 153 length_67064	109	CRISPR start position: 22,417 ––––– CRISPR end position: 22,526
				DR consensus: CCGGATAAGCAAAGCGCATCCGGCA
				DR length: 25 Number of spacers: 1
CRISPR	crispr_1	NODE 47 length_29973	146	CRISPR start position:29,879 ––––– CRISPR end position: 30,025 DR consensus: TTTGAGGTGTACTGGCAATAGCGGACACTACCATTTGTTCTTTTTTTAAGCAG DR length: 53 Number of spacers: 1
Cas cluster	General-Class1			Start 11,924 End 13,075; Gene name Cas3_1_I, Orientation ( +)

#### Proteomic characterization

**Fig. 4:**
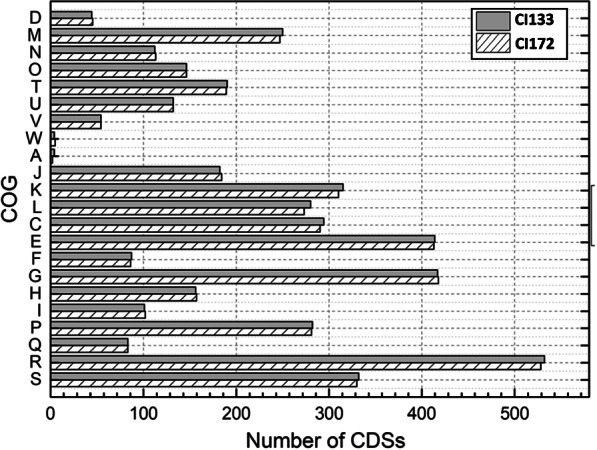
CDSs corresponding to the COG functional categories represented in the CI133 and CI172 strains genomes. The letters represent each one of the functional categories: [D] Cell cycle control, cell division, chromosome partitioning; [M] Cell wall/membrane/envelope biogenesis; [N] Cell motility; [O] Post-translational modification, protein turnover, and chaperones; [T] Signal transduction mechanisms; [U] Intracellular trafficking, secretion, and vesicular transport; [V] Defense mechanisms; [W] Extracellular structures; [A] RNA processing and modification; [J] Translation, ribosomal structure and biogenesis; [K] Transcription; [L] Replication, recombination and repair; [C] Energy production and conversion; [E] Amino acid transport and metabolism; [F] Nucleotide transport and metabolism; [G] Carbohydrate transport and metabolism; [H] Coenzyme transport and metabolism; [I] Lipid transport and metabolism; [P] Inorganic ion transport and metabolism; [Q] Secondary metabolites biosynthesis, transport, and catabolism; [R] General function prediction only; [S] Function unknown

In concordance with the genomic analysis, a similarity was observed between the proteomes of both strains, 4,540 proteins were identified and quantified by LC/MS–MS using Label Free Quantification (LFG). As shown in Fig. 4, the Clusters of Orthologous Groups of proteins (COGs) assignment did not show great differences between the CI133 and CI172 strains [41]. The COG categories more represented in both strains were the carbohydrate transport and metabolism (G), the amino-acids transport and metabolism (E), those related to poorly characterized proteins with a general function prediction (R) and of unknown function (S) (Fig. 4).

However, when the proteomic results determined in a defined medium simulating the host`s environment were more deeply analyzed, we observed the presence of specific proteins in one or another of the strains under study. Thus, 16 of the 4,540 proteins were specifically expressed in CI133 but not detected in the CI172 strain (Table 9). In the same way, 12 of the total determined proteins were only expressed in the CI172 strain (Table 9).

**Table 9:** Experimental proteins detection in CI133 or CI172 strains

**Accession ID**^a^	**Gene**	**Description**	**Coverage (%)**	**Number of matching peptides**	**MW (kDa)**	**Calculated IP**	**COG**^b^
**CI133 strain**							
Q83SN2	*secA*	Protein translocase subunit SecA OS = *Shigella flexneri* OX = 623 GN = *secA* PE = 3 SV = 1	16.98	44	102	5.6	U
Q821A7	*pssA*	Phosphatidylserine synthase OS = *Shigella flexneri* OX = 623 GN = *pssA* PE = 4 SV = 2	22.39	20	52.8	9.07	I
Q83R07	SF301_0227/SF2075	Putative enzyme of sugar metabolism OS = *Shigella flexneri* OX = 623 GN = SF2075 PE = 4 SV = 1	38.69	21	29.7	5.62	M/G
Q83RW2	*appA*	Phosphoanhydride phosphorylase pH 2.5 acid phosphatase OS = *Shigella flexneri* OX = 623 GN = *appA* PE = 4 SV = 4	24.54	15	47.1	6.35	S
Q83R69	*ptrB*	Protease II OS = *Shigella flexneri* OX = 623 GN = ptrB PE = 4 SV = 1	9.77	10	79.4	6.04	E
P60788	*lepA*	Elongation factor 4 OS = *Shigella flexneri* OX = 623 GN = l*epA* PE = 3 SV = 1	25.21	24	66.5	5.59	M
Q7UBC6	*plsB*	Glycerol-3-phosphate acyltransferase OS = *Shigella flexneri* OX = 623 GN = *plsB* PE = 3 SV = 1	10.16	19	93.7	8.51	I
P59609	*argG*	Argininosuccinate synthase OS = *Shigella flexneri* OX = 623 GN = *argG* PE = 3 SV = 2	30.43	27	49.9	5.39	E
P0A9K0	*pheA*	Bifunctional chorismate mutase/prephenate dehydratase OS = *Shigella flexneri* OX = 623 GN = *pheA* PE = 3 SV = 1	25.91	18	43.1	6.68	E
Q83LN3	*pncB*	Nicotinate phosphoribosyltransferase OS = *Shigella flexneri* OX = 623 GN = *pncB* PE = 3 SV = 4	16.75	15	45.9	6.7	H
Q83SM9	*guaC*	GMP reductase OS = *Shigella flexneri* OX = 623 GN = g*uaC* PE = 3 SV = 1	13.54	17	37.4	6.54	F
A0A0H2UYQ5	*acrB*	Efflux pump membrane transporter OS = *Shigella flexneri* OX = 623 GN = *acrB* PE = 3 SV = 1	6.20	9	113.6	5.63	V
Q83JU0	*visC*	Uncharacterized protein OS = *Shigella flexneri* OX = 623 GN = *visC* PE = 4 SV = 1	8.25	4	44.2	7.06	S
P0ABN8	*dcuA*	Anaerobic C4-dicarboxylate transporter DcuA OS = *Shigella flexneri* OX = 623 GN = *dcuA* PE = 3 SV = 1	2.31	4	45.7	7.75	C
Q83PJ4	*asnA*	Aspartate–ammonia ligase OS = *Shigella flexneri* OX = 623 GN = *asnA* PE = 3 SV = 1	26.67	9	36.6	5.87	E
Q83QV4	*ccmF*	Cytochrome c-type biogenesis protein OS = *Shigella flexneri* OX = 623 GN = *ccmF* PE = 4 SV = 1	3.55	4	71.3	9.63	O/C
**CI172 strain**							
A0A0H2UZT3	*yncB*	Putative oxidoreductase OS = *Shigella flexneri* OX = 623 GN = *yncB* PE = 4 SV = 1	25.13	42	43.5	6.54	C/R
P0AGE8	*chrR*	Quinone reductase OS = *Shigella flexneri* OX = 623 GN = *chrR* PE = 3 SV = 1	72.87	34	20.4	5.15	R
P0A733	*mgsA*	Methylglyoxal synthase OS = *Shigella flexneri* OX = 623 GN = *mgsA* PE = 3 SV = 1	32.89	4	16.9	6.64	G
Q83K78	*pdxK*	Pyridoxine/pyridoxal/pyridoxamine kinase OS = *Shigella flexneri* OX = 623 GN = *pdxK* PE = 3 SV = 1	26.86	25	30.9	5.34	H
P64466	*ydfZ*	Putative selenoprotein YdfZ OS = *Shigella flexneri* OX = 623 GN = *ydfZ* PE = 3 SV = 1	26.87	8	7.3	8.21	C/E
P0ADZ6	*rpsO*	30S ribosomal protein S15 OS = *Shigella flexneri* OX = 623 GN = *rpsO* PE = 3 SV = 2	49.44	11	10.3	10.4	J
Q83JU4	*dsbC*	Thiol:disulfide interchange protein OS = *Shigella flexneri* OX = 623 GN = *dsbC* PE = 3 SV = 1	21.19	10	25.6	6.79	O
Q83PE8	*fdhE*	Protein FdhE homolog OS = *Shigella flexneri* OX = 623 GN = *fdhE* PE = 3 SV = 1	9.06	7	34.7	5.35	O
Q83ME5	*panB*	3-methyl-2-oxobutanoate hydroxymethyltransferase OS = *Shigella flexneri* OX = 623 GN = *panB* PE = 3 SV = 1	17.05	5	28.2	5.78	H
Q83J34	*xylB*	Xylulose kinase OS = *Shigella flexneri* OX = 623 GN = *xylB* PE = 3 SV = 1	9.92	6	52.6	5.8	G
P0A7F2	*pyrH*	Uridylate kinase OS = *Shigella flexneri* OX = 623 GN = *pyrH* PE = 3 SV = 2	35.27	8	26	7.39	F
Q83SP1	*leuB*	3-isopropylmalate dehydrogenase OS = *Shigella flexneri* OX = 623 GN = *leuB* PE = 3 SV = 3	11.29	4	39.5	5.38	E

^a^Accession number of Uniprot database

^b^Clusters of Orthologous groups (COG): U-Intracellular trafficking, secretion, and vesicular transport; I-Lipid transport and metabolism, M-Cell wall/membrane/envelope biogenesis, G-Carbohydrate transport and metabolism, E-Amino acid transport and metabolism, H-Coenzyme transport and metabolism, F-Nucleotide transport and metabolism, V-Defense mechanisms, C-Energy production and conversion, O-Posttranslational modification, protein turnover, chaperones, R-General function prediction only, J-Translation, ribosomal structure and biogenesis, S-Function unknown

Moreover, significant differences (*p* < 0.05) in the expression of a total of 33 proteins were also detected, 26 of which were 1 to threefold upregulated in CI133 respect to CI172 (Table 10, positive values), while the last 7 proteins were overexpressed in CI172 respect to CI133 (Table 10, negative values).

**Table 10:** Differential proteins expression between CI133 and CI172 strains

**Accession ID**^a^	**Gene**	**Description**	**Coverage (%)**	**Number of matching peptides**	**MW (kDa)**	**Calculated IP**	**COG**^b^	**Fold Change CI133 vs CI172**	**p-value**
Q83QS6	*nuoC*	NADH-quinone oxidoreductase subunit C/D OS = *Shigella flexneri* OX = 623 GN = *nuoC* PE = 3 SV = 1	14.00	18	68.7	6.42	C	3.0	0.0085
Q83Q57	*yqhD*	Putative oxidoreductase OS = *Shigella flexneri* OX = 623 GN = *yqhD* PE = 4 SV = 4	45.22	54	42.1	6.13	R	3.0	0.0315
P0A247	*virB*	Virulence regulon transcriptional activator VirB OS = *Shigella flexneri* OX = 623 GN = *virB* PE = 1 SV = 1	37.54	45	35.4	9.55	U	2.8	0.0374
A0A0H2UZ50	*asnB*	Asparagine synthetase B OS = *Shigella flexneri* OX = 623 GN = *asnB* PE = 4 SV = 1	46.80	70	58.4	6.06	E	2.7	0.0365
A0A0H2UWX4	*thrC*	Threonine synthase OS = *Shigella flexneri* OX = 623 GN = *thrC* PE = 4 SV = 1	46.03	52	47.2	5.34	E	2.5	0.0399
Q83L32	*ppsA*	Phosphoenolpyruvate synthase OS = *Shigella flexneri* OX = 623 GN = *ppsA* PE = 3 SV = 1	26.64	66	87.4	5.06	G	2.3	0.0208
Q7BU69	*virA*	Cysteine protease-like VirA OS = *Shigella flexneri* OX = 623 GN = *virA* PE = 1 SV = 1	43.50	35	44.7	6.11	U	2.2	0.0457
P0A1I5	*mxiA*	Protein MxiA OS = *Shigella flexneri* OX = 623 GN = *mxiA* PE = 1 SV = 1	17.49	27	76.1	5.26	U	2.2	0.0291
Q83S97	*sucD*	Succinate–CoA ligase [ADP-forming] subunit alpha OS = *Shigella flexneri* OX = 623 GN = *sucD* PE = 3 SV = 1	24.22	17	29.7	6.79	C/H	2.0	0.0414
Q83M53	*ybaU*	Peptidylprolyl isomerase OS = *Shigella flexneri* OX = 623 GN = *ybaU* PE = 4 SV = 1	27.93	37	68.1	5.11	M	1.9	0.0319
P0A1C1	*sctN*	Probable ATP synthase SpaL/MxiB OS = *Shigella flexneri* OX = 623 GN = *spaL* PE = 1 SV = 1	22.09	22	47.5	5.68	Q	1.9	0.0474
Q83K88	SF2445	Putative aminotransferase OS = *Shigella flexneri* OX = 623 GN = SF2445 PE = 4 SV = 4	29.37	40	46.1	7.34	S	1.8	0.0475
P0A943	*bamA*	Outer membrane protein assembly factor BamA OS = *Shigella flexneri* OX = 623 GN = *bamA* PE = 2 SV = 1	28.52	40	90.5	5.12	M	1.8	0.0156
Q83S94	*sdhB*	Succinate dehydrogenase iron-sulfur subunit OS = *Shigella flexneri* OX = 623 GN = *sdhB* PE = 3 SV = 1	10.50	6	26.8	6.73	C/O	1.7	0.0253
P0A3B4	*bipA*	GTP-binding protein TypA/BipA OS = *Shigella flexneri* OX = 623 GN = *typA* PE = 3 SV = 1	26.52	57	67.3	5.33	T	1.7	0.0399
P63738	*carB*	Carbamoyl-phosphate synthase large chain OS = *Shigella flexneri* OX = 623 GN = *carB* PE = 3 SV = 2	21.62	67	117.8	5.34	E/F	1.7	0.0473
Q83J38	*glyS*	Glycine–tRNA ligase beta subunit OS = *Shigella flexneri* OX = 623 GN = *glyS* PE = 3 SV = 1	35.12	70	76.7	5.44	J	1.6	0.0296
P0AAI4	*ftsH*	ATP-dependent zinc metalloprotease FtsH OS = *Shigella flexneri* OX = 623 GN = *ftsH* PE = 3 SV = 1	25.78	43	70.7	6.24	D	1.6	0.0381
Q83PZ8	*yhdH*	Putative dehydrogenase OS = *Shigella flexneri* OX = 623 GN = *yhdH* PE = 4 SV = 1	37.04	38	34.7	5.91	S	1.5	0.0411
P0AAC3	*uspE*	Universal stress protein E OS = *Shigella flexneri* OX = 623 GN = *uspE* PE = 3 SV = 2	50.32	52	35.7	5.31	V	1.4	0.0149
Q83PF3	*hemN*	Coproporphyrinogen-III oxidase OS = *Shigella flexneri* OX = 623 GN = *hemN* PE = 3 SV = 4	12.69	15	52.8	6.27	H	1.3	0.0497
Q83S93	*sdhA*	Succinate dehydrogenase flavoprotein subunit OS = *Shigella flexneri* OX = 623 GN = *sdhA* PE = 3 SV = 4	12.07	27	64.4	6.23	C/O	1.2	0.0022
P0ADE9	*ygfZ*	tRNA-modifying protein YgfZ OS = *Shigella flexneri* OX = 623 GN = *ygfZ* PE = 3 SV = 2	14.72	16	36.1	5.27	O	1.2	0.0282
Q83QP0	*nupC*	Nucleoside permease OS = *Shigella flexneri* OX = 623 GN = *nupC* PE = 3 SV = 4	12.25	13	43.5	8.48	F	1.2	0.0158
P0AA21	*ompR*	Transcriptional regulatory protein OmpR OS = *Shigella flexneri* OX = 623 GN = *ompR* PE = 3 SV = 1	23.85	18	27.3	6.39	K/T	1.2	0.0093
P0A9V4	*lptB*	Lipopolysaccharide export system ATP-binding protein LptB OS = *Shigella flexneri* OX = 623 GN = *lptB* PE = 3 SV = 2	14.11	11	26.8	5.99	M/N	1.0	0.0366
P0A958	*eda*	KHG/KDPG aldolase OS = *Shigella flexneri* OX = 623 GN = *eda* PE = 3 SV = 1	76.53	72	22,3	5.67	M	-1.1	0.0257
Q83J15	*pyrE*	Orotate phosphoribosyltransferase OS = *Shigella flexneri* OX = 623 GN = *pyrE* PE = 3 SV = 4	60.09	34	23.5	5.48	F	-1.2	0.0469
Q83LA7	*ycjY*	Uncharacterized protein OS = *Shigella flexneri* OX = 623 GN = *ycjY* PE = 4 SV = 1	33.66	39	33.7	5.02	S	-1.3	0.0455
Q83SP4	*tbpA*	Thiamin-binding periplasmic protein OS = *Shigella flexneri* OX = 623 GN = *tbpA* PE = 4 SV = 4	15.90	24	36.2	7.72	H	-1.3	0.0129
Q83PR9	*dppA*	Dipeptide transport protein OS = *Shigella flexneri* OX = 623 GN = *dppA* PE = 4 SV = 1	38.69	96	60.3	6.65	H	-1.5	0.0112
P0AFX3	*hpf*	Ribosome hibernation promoting factor OS = *Shigella flexneri* OX = 623 GN = *hpf* PE = 3 SV = 1	25.26	13	10.7	7.05	V	-1.8	0.0273
P66608	*rpsG*	30S ribosomal protein S7 OS = *Shigella flexneri* OX = 623 GN = *rpsG* PE = 3 SV = 2	43.59	67	17.6	10.3	J	-1.9	0.0310

^a^Accession number of Uniprot database

Positive value: upregulated in CI133; Negative value: upregulated in CI172

^b^Clusters of Orthologous groups (COG): R-General function prediction only, U-Intracellular trafficking, secretion, and vesicular transport, E-Amino acid transport and metabolism, G-Carbohydrate transport and metabolism, C-Energy production and conversion, H-Coenzyme transport and metabolism, M-Cell wall/membrane/envelope biogenesis, Q-Secondary metabolites biosynthesis, transport and catabolism, J-Translation, ribosomal structure and biogenesis, T-Signal transduction mechanisms, F-Nucleotide transport and metabolism, D-Cell cycle control, cell division, chromosome partitioning, V-Defense mechanisms, O-Posttranslational modification, protein turnover, chaperones, K-Transcription, N-Cell motility, S-Function unknown

**Fig. 5:**
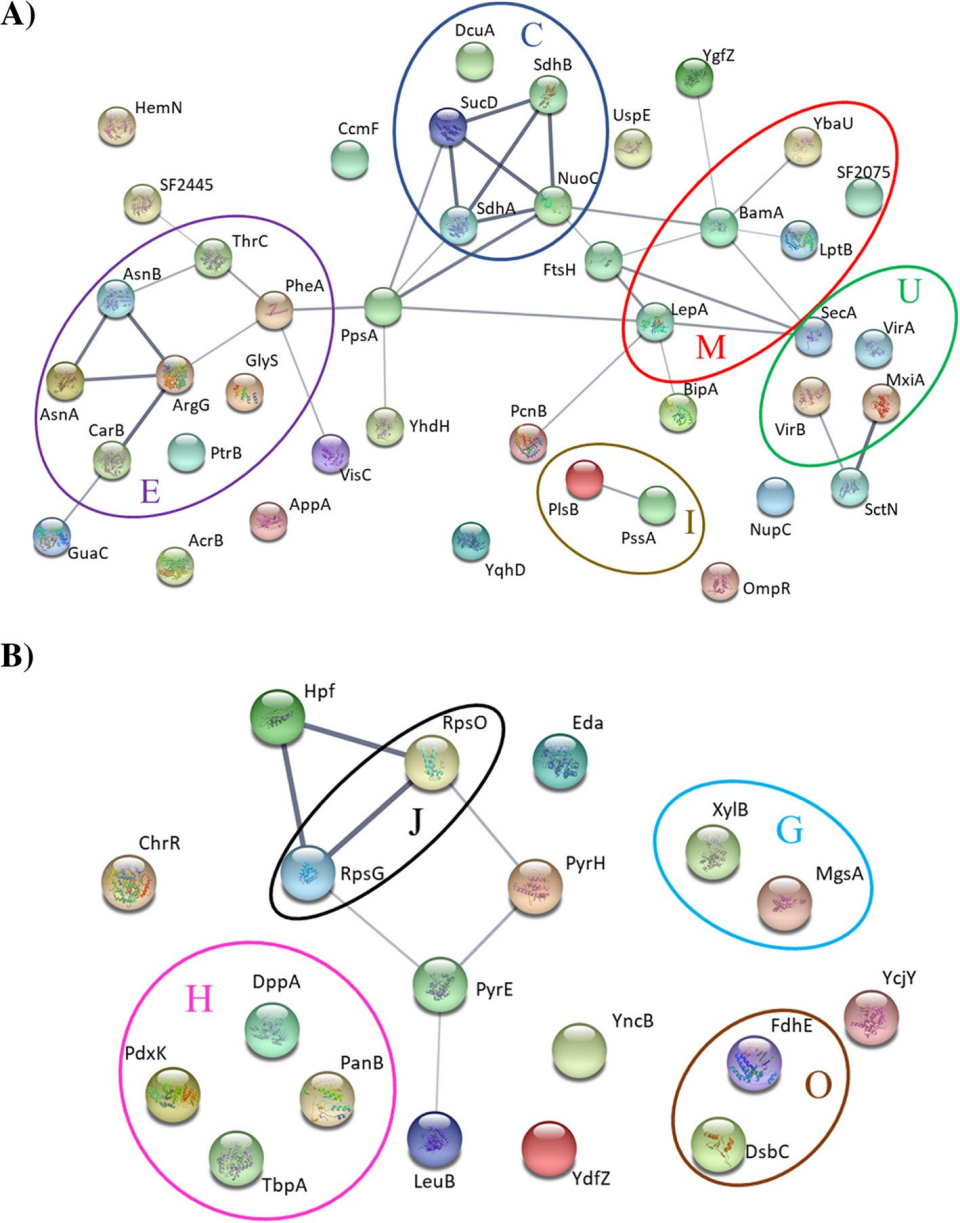
Protein–Protein interactive networks. **A** Proteins present and upregulated in the CI133 strain. **B** Proteins showing interactions present in the CI172 strain. The proteins are represented with the nodes, the edges represent the interactions between the proteins, and the width of edges the degree of interactions. The circles are showing different COG categories: [O] Post-translational modification, protein turnover, and chaperones; [U] Intracellular trafficking, secretion, and vesicular transport; [C] Energy production and conversion; [E] Amino acid transport and metabolism; [M] Cell wall/membrane/envelope biogenesis; [I] Lipid transport and metabolism; [J] Translation, ribosomal structure and biogenesis; [H] Coenzyme transport and metabolism and [G] Carbohydrate transport and metabolism. The networks were constructed using the STRING v11.5 bioinformatics tool

To understand the relation and the involvement of these proteins with a particular process or function we constructed protein networks using STRING v11.0 with the proteins expressed only in CI133 or CI172 plus the upregulated proteins in each strain (Tables 9 and 10). For S.* flexneri* 2 CI133 strain, the 42 proteins, taking in account (16 only present and 26 upregulated in this strain), represented by the nodes showed 37 edges or interactions (Fig. 5A). The number of nodes determined was significantly higher than the calculated by STRING for a random set of proteins, suggesting that these proteins could have a biological connection, as a group. The main represented functional categories were the metabolism and transport of amino-acids (E), energy production and conversion (C), intracellular trafficking, secretion, and vesicular transport (U) and cell wall/membrane/envelope biogenesis (M) (Fig. 5A). Meanwhile, for the CI172 strain, were only found 7 edges among the 19 proteins (12 only present and 7 upregulated, respectively) (Fig. 5B). The most represented category was coenzyme transport and metabolism (H). However, the proteins included in category translation, ribosomal structure and biogenesis (J) are the ones that showed the greatest number of interactions between themselves and between proteins from other categories (Fig. 5B).

These set of proteins that were found either overexpressed or only present in one of the two strains (Tables 9 and 10) were most of them functionally associated to cellular metabolism. However, between the proteins that were overexpressed in CI133 we identified the virulence regulon, including the transcriptional activator VirB, the VirA protease, the SecA translocase and the MxiA protein. VirB, encoded in *Shigella*’s virulence plasmid, is a master regulator of a great number of genes including those required for synthesis and formation of the Type Tree Secretion System (T3SS) containing *mxiA* [42–44]. MxiA, is an essential membrane protein involved in the secretion process of T3SS’s substrates [45, 46]. VirA protein is a virulence effector, translocated into the host by the T3SS, to produces the host cell membrane ruffling necessary for the pathogen entry and for intra and intercellular spreading [47]. The SecA translocase subunit protein is a part of the secretion system SecAB also involved in *Shigella’s* virulence [48].

Therefore, despite we were able to see similarities between the proteomes expressed in both strains, we were able to determine differential expression of 33 proteins. According to the differences found in the genomes and in the differential expression of proteins, we can suggest that each of the analyzed strains can be adapted differently to the host environment to cause infection.

## Discussion

Previous report demonstrated that *S. flexneri* is the endemic pathogen in most developing countries, including Argentina, being the serovars 1b, 2a, 3a, 4a and 6 predominant, while *S. flexneri* serovar 2a, as *S. sonnei*, is frequently isolates in industrialized countries [49]. In accordance, in our previous work we have demonstrated that *S. flexneri* 2a is the gastroenteric infectious agent with the highest incidence in the NWA region, representing the main cause of children’s morbidity and mortality [19]. However, we also demonstrated that the prevalence of this serotype was observed in the last 7 years, since in the period from at least 2007 to 2013 the *S. flexneri* AA479 serovar cause the most gastroenteric disease in the NWA population [19]. Due to the continuous turnover of serovars observed, in this work we performed the genomic and proteomic analysis of two clinical isolates obtained in different periods of time, with a four-year of difference between one from the other. Our objective was to detect molecular changes that may have occurred during this period, which could help us understand or explain the potential establishment and persistence of this pathogen in the population under study.

The data obtained allowed us to determine the pangenome, the “core” genome and the variable genome of the 80 available strains as well as our CI133 and CI172 clinical isolates. We observed that the “core” genome here described represents most of the analyzed *S*. *flexneri* 2 genomes, and it is relatively conserved. These results are in agreement with data already published by numerous authors, supporting the idea that *Shigella* has a conserved “core” genome in which the virulence determinants (that allow it to colonize, persist and spread in the region) are perhaps encoded [50–52].

When the variable genome was analyzed, we observed that the isolates from the NWA region presented a large number of unique genes, suggesting that this serovar evolved in recent years through the acquisition of these strain-specific genetic elements. The results obtained in this work are also in agreement with those reported by Shen et al., who analyzed the pangenome of 15 *S. flexneri* isolates and identified a number of specific genes comparable to those obtained in this study [52]. It is important to note that, even though the CI172 strain showed a lower number of strain-specific genes, we observed a greater number of these genes with known function than those obtained in the CI133 strain (24 of 74 vs. 29 of 112, respectively). Among the specific genes of the CI133 strain, the presence of *mdtL* and *yeaN* is noteworthy. These genes are both part of the Major Facilitator Superfamily (MFS) composed by thousands of transport proteins that are well characterized [53]. MFS are involved in the transport of a great variety of small molecules such as sugars, amino acids, and drugs [54]. Interestingly, some of them are specifically related to Multi Drug Resistance (MDR), known as MFS-MDR transporters [55, 56]. MFS-MDR can carry out an active flux of drugs, antibiotics, or toxins through a cation/substrate antiport mechanism generating resistance to them. Furthermore, there are some reports indicating that the *yeaN* gene product could be acting as a 2-nitroimidazol transporter conferring *E. coli* resistance to this drug [57]. On the other hand, among the specific genes identified in CI172 genome, we were able to determine the presence of the complete set of genes that codifies for the production, immunity and lysis protein of the colicin E2 and Rel toxin-antitoxin systems. Colicins are high molecular weight toxic proteins that are produced by colicinogenic *E. coli* strains and some related species of the *Enterobacteriaceae* family [58–61], being at least 23 colicin types have been described in detail [62]. Colicin production has been well described among *Shigella* genera, especially for the subspecies *S*. *sonnei* [63]. Interestingly, from the total of 82 genomes analyzed in this study Colicin E codification system was only found in the CI172 strain isolated in this work (Table S2). Besides, we identified the neighbor genes as plasmid related genes suggesting a plasmidic origin or that CI172 could have acquired this bacteriocin through plasmid transference. Although MvpAT is the most commonly described antitoxin system (TA) in *S. flexneri*, we were unable to identify these genes in either genome, instead RelEB-TA was identified among the CI172-specific genes. The RelEB-TA systems usually consist of two genes that encode a stable toxin and an unstable antitoxin that inhibits the toxin [64]. RelE toxin is known to be involved in the emergence of persistent strains, resistant to multiple drugs, biofilm formation, and regulation of the bacterial population by inducing dormancy [65–67]. This TA system is present in the virulence plasmid and its main function is to ensure its stability in bacteria [68, 69]. However, in the CI172 strain this TA system was located within the NODE_575_length_1729, which was not associated with plasmid regions. The homology search of the complete node-575 sequence by BLASTn, it was observed that the TA system coding region is flanked upstream by the IS600 element (NP_707432.1) and downstream by the gene coding for SF1546 a bacteriophage protein (NP_707877.2). These data suggest that the CI172 strain acquired the TA system into its genome by mobile genetic elements such as insertion sequences and phages. This could explain the multiple antibiotic resistances phenotype of CI172 strain determined “in vitro”. Therefore, we suggest that the presence of these genes could be addressed as part of the acquired adaptive advantages allowing the persistence of *S. flexneri* serotype 2 in the NWA region. Together the pangenomic, phylogenetics strain-specific genes and plasmid profile analysis show that strain CI172 is quite singular, unique and different from the rest of the studied strains. Furthermore, the phylogenetic analysis reveals that both strains studied in this work, isolated in Argentina, are closely related to those isolated in Europe than to those from other continents.

Multi-drug resistance has been highly addressed in the last decades, antibiotics resistance spread is a worldwide worrisome. Most of *Shigella*’s serotypes are not susceptible to first-generation antibiotics, such as ampicillin, tetracycline, sulfonamides, chloramphenicol, nalidixic acid, and Trimethoprim/sulfamethoxazole. Therefore, last-generation antibiotics, such as fluoroquinolones are preferred. However, these are more expensive and resistance to them are starting to show up in the countries with more consume [70, 71]. The “in silico” analysis of the two genome sequences using CARD and ResFinder databases allowed us to determine the presence of several genes that encode antibiotic resistance which was then experimentally confirmed, demonstrating that the strains under study are presented with an MDR phenotype. Despite this, both strains were differentiated mainly by the presence of the *marA* gene, which was identified in CI133 but not in CI172. The *marA* gene was previously reported to provide multiple drug resistance [72]. Has been proposed that the transcription factor MarA determines a multidrug resistance phenotypes, allowing for the resistance of quinolones, tetracyclines and fluoroquinolones by modulating efflux pump and porin expression [72]. The presence of this gene in CI133 suggested a broad spectrum of resistance; however CI172 was additionally resistant to trimethoprim/sulfamethoxazole. At the beginning, shigellosis was used to be treated with antibiotics such as sulphonamides, tetracycline, and chloramphenicol [73]. The emerging resistance to those antibiotics enforced the use of additional drugs such as ampicillin, co-trimoxazole, nalidixic acid, and fluoroquinolones to combat the infection. Chloramphenicol, ampicillin, and tetracycline resistances that were found in CI133 and CI172 are in concordance with the resistance phenotypes already reported [50]. However, vancomycin and imipenem resistances present in both strains have not been between the most common antibiotic resistance phenotypes among *Shigella* strains. The family of carbapenemases enzymes, by plasmid encoded, is one of the most significant health challenges of the century, given the potential for dissemination between species and mortality rates due to infections caused by bacteria. The emerging resistance to carbapenems represents a serious concern since these drugs are often the last line of effective therapy available for the treatment of infections caused by MDR bacteria [74–76]. However, the pathogens persistence is not only result of MDR, but also of the adaptative advantages acquired and its virulence performance.

Using VirulenceFinder we identified 10 of the main genes involve in *Shigella*’s pathogenesis in different contigs of the CI133 strain and 11 on the CI172 strain, displaying a new difference between these two isolates. In addition, when the homology analysis against plasmid pCP301 was performed we identified other virulence factors not detected by the program. The results obtained allowed us to determine those genes that may be forming part of the bacterial chromosome, while others are forming part of the virulence plasmid pCP301 [29]. Among the genes identified by the mentioned software, it is noteworthy that *sigA*, *pic*, *lpfA*, *gad* and *sitA* would be located on both genomes. In concordance with our localization, Henderson et al. (1999) reported that in enteroaggregative *E. coli* (EAEC) 042 and *S. flexneri* 2457 T the *pic* gene is encoded within the chromosomes [77]. Interestingly, in CI133 and CI172, *pic* and *sigA* genes were located in same contigs, thus being able to affirm the chromosomal location of both. Moreover, the products of these genes are member of the subfamily of autotransporter proteins termed the SPATEs (serine protease autotransporters) of *Enterobacteriaceae* [77, 78]. While, the *lpfA*, *gad* and *sitA* genes products are involved in the major fimbriae formation, the controls of bacterial acid resistance-SlyA dependent and in an iron-ABC transport system during the eukaryotic cells infection, respectively [79–81].

Numerous mobile elements can be found in the variable genome of a strain, which are the result of evolution by selection of genes acquired by horizontal transfer (THG). The determination of the mobilome (set of mobile elements) in a genome allows characterizing the sequenced genomes. The mobilome can be constituted by integrative and conjugative elements, prophages, and plasmids. The CRISPRs systems and bacteriocin genes also contribute to the diversity of the accessory genome. The use of services available in the Center for Genomics Epidemiology and in different web sites, as well the combination of the results obtained from each one, we were able to identify the mobilome of the strain under study. As above mentioned, we could determine that numerous virulence genes are contained in pCP301, a plasmid previously described as essential for establishing infection in the host. This plasmid is a member of the IncFII incompatibility group, element also present in the sequence of CI133 and CI172 [29]. In addition to the virulence genes and the IncFII element, we were able to identify the contigs of the analyzed sequences harboring those genes that code for the replication control, stability and mobilization of the plasmid, as well as several insertion sequences that showed homology with pCP301 [29]. This allowed us to conclude that this plasmid is responsible for the establishment of the infection of CI133 and CI172 pathogenic strains.

On the other hand, detection of Col156 and ColRNAI into CI172 sequence showed two more major differences with the CI133 strain. Col156 is the genetic element that characterizes plasmids harboring the synthesis, immunity and lysis system of colicin E, which was identified in contig 16 of CI172 as well as the *ceaB* synthesis genes, the immunity *ceiB* and the lysine *celB* of the colicin E2 system [30]. The homologues search of this contigs sequence showed 99% identity with pColE2-P9 of about 7 Kb of *Shigella*, which occurs in low number of copies per chromosome [31–33]. These data correlate with our experimental results, where we were able to detect a band that migrate approximately at that size and that stains poorly with ethidium bromide. Similarly, “in silico” and experimentally, we demonstrate the presence of pSF301-3 in the CI172 strain, more precisely in contig 40. Like pColE2-P9, pSF301-3 has the ability to be mobilized when a conjugative plasmid is harbored within the same strain [37], indicating that both strains in this study could have evolved in a period of four years through the acquisition of these mobile elements.

The presence of prophages on the chromosome can allow some bacteria to acquire resistance to antibiotics, exist in new environmental niches, even improve adhesion or become pathogens. Thus, the numerous phage regions found in the CI133 and CI172 strains could be related to the MDR phenotype presented by these strains, although the correlation between both factors analyzed could not be precisely established. This suggestion is supported by the numerous reports describing the joint detection of resistance to TMS and ampicillin related to integrons present in phages [82]. Due to the presence of phages, in these strains the presence of CRSIPR elements was also analyzed, since these represent a type of immune system of the bacterium to resist the attack of phages [83, 84]. As expected, numerous regions were found that could be part of these elements in both strains, highlighting the importance of the CRISPR-Cas systems, where the Cas proteins involved in the mechanism of immunity against phages could be identified. Even though the relationship between prophages and CRISPR has not been determined, numerous experimental studies are being carried out in our laboratory to determine its importance in the evolution of the prevalent strains in the region.

To complete the genomic study, the secretome of both strains was also analyzed in a medium simulating the environment of the host. In total, a great difference was not observed between both strains in the number of proteins that were synthesized and exported to the culture medium or when the Clusters of Orthologous Groups of proteins was determined. In both proteomes the great majority of the proteins identified by LFQ were the carbohydrate transport and metabolism, the amino acid transport and metabolism, the transcription and the cell wall/membrane/envelope biogenesis. Interestingly, despite the similarities observed, it was possible to detect proteins differentially present in one or another strain, as well as proteins that were overexpressed in one isolate with respect to the other. In addition, in the CI133 strain a greater number of proteins capable of interacting were observed respect to the interactions obtained in the CI172 strain. This observation could be due to the greater number of proteins used in the analysis. However, these differences suggest that both strains have undergone different evolutionary processes for host adaptation and infection. We here suggest that this evolutionary process is more efficient in the CI172 strain since a lower number of secreted proteins would have a lower energy cost for the bacteria but would not affect its pathogenesis.

## Conclusion

In summary, even when great similarities were observed between the CI133 and CI172 strains and those other from different countries, confirming the high percentage of genes constituting the core genome of *S. flexneri* 2. In this work a considerable number of strain specific genes were identified. On the bases of these observations, we can address the importance of characterizing rapid spread of pathogenic bacteria in specific region. We here hypothesize that the identification of these highly identical or strains specific genomic and/or proteomic factors into other isolated of our collation allow us to develop molecular markers that are characteristic of the NWA region circulating pathogens. In addition, the most innovative result of this work is, to our knowledge, the first description of a producer of *S. flexneri* 2 Colicin E, a factor recently acquired by mobile elements (horizontal gene transfer), as one of the characteristics that allow *S. flexneri* 2 persist in the microbial community. It is important to highlight that, like the CI172 strain, at least 350 isolates of *Shigella* producing antimicrobial compounds have been also previously identified in our laboratory. This supports our hypothesis that the production of these compounds has been widely distributed among the circulating pathogens in the region under study.

## Methods

### Strain information

The clinical isolates of *S. flexneri* 2 (CI133 and CI172), were isolated from female children patient with severe acute clinical manifestations of shigellosis in the NWA region. The CI133 and CI172 strains were isolated from children patients suffering diarrhea [19]. The strains were routinely grown at 37 °C overnight on LB to stationary phase. These strains were identified and characterized in a previous work [19].

### Genome sequencing and assembly

DNA extraction and WGS sequencing of the CI133 and CI172 strains were performed by Macrogen Humanizing Genomics (Seoul, Korea), using the Illumina HiSeq2500 platform of paired-end libraries, taking as reference the sequence of strain *S. flexneri* 2a str. 301 from the NCBI database (access number NC_004337). The quality of the raw data obtained from the sequencing of these genomes was analyzed with the FastQC program (https://www.bioinformatics.babraham.ac.uk/projects/fastqc/). The assembly was made through Velvet 1.2.1, using the best k-mer value with a value of k = 81 for the CI133 strain, and k = 79 for the CI172 strain [85]. Genome annotation was performed according to standard procedures described by Prokaryotic Genome Annotation Pipeline (PGAP) from NCBI (https://www.ncbi.nlm.nih.gov/genome/annotation_prok/). The pangenomic analysis of the 80 strains of *Shigella flexneri* 2 plus the two newly sequenced strains (CI133 and CI172) were performed through the Microscope Platform with an identity of 80% and a coverage of 80% [86].

### Data availability

The Whole Genome Shotgun project of *Shigella flexneri* 2 CI133 strain has been deposited at DDBJ/ENA/GenBank under the accession JAGDQG000000000, the Bioproject accession number is PRJNA498014 and the Biosample accession number is SAMN10275217. While the Whole Genome Shotgun project of *Shigella flexneri* 2 CI172 strain has been deposited at DDBJ/ENA/GenBank under the accession JAGDQF000000000, the Bioproject accession number is PRJNA498020 and BioSample accession number is SAMN10275218. The versions described in this paper are the versions JAGDQG010000000 and JAGDQF010000000 for the CI133 and CI172 strains, respectively.

### Phylogenetic analysis

A multi locus sequence typing (MLST) was performed using fifteen housekeeping genes, including *arcA* (DNA-binding transcriptional dual regulator), *aroE* (shikimate dehydrogenase), *aspC* (aspartate aminotransferase), *clpX* (ATP-dependent Clp protease ATP-binding subunit), *cyaA* (adenylate cyclase), dNaG (DNA primase), *fadD* (fatty acyl-CoA synthetase), *grpE* (nucleotide exchange factor), *icdA* (isocitrate dehydrogenase), *lysP* (lysine:H( +) symporter), *mdh* (malate dehydrogenase), *mtlD* (mannitol-1-phosphate 5-dehydrogenase), *mutS* (DNA mismatch repair), *rpoS* (RNA polymerase, sigma 38 factor) and *uidA* (beta-glucuronidase), as previously described [87]. The concatenated sequences were align using MUSCLE (https://doi.org/10.1093/nar/gkh340) and the phylogenetic tree was inferred with UPGMA method and constructed with MEGAX [88].

### Antibiotic resistance genes, virulence factors and mobile elements prediction

Antibiotic resistance genes were identified using The Comprehensive Antibiotic Resistance Database CARD (http://arpcard.mcmaster.ca) [89]. The presence of genetic determinants of drug resistance and mobile elements in the complete sequence of CI133 or CI172 was also investigated using those serves of the Center for Genomics Epidemiology. For antibiotic resistance genes identification we used ResFinder-4.1 server (https://cge.cbs.dtu.dk/services/ResFinder/) [90]. As selection criteria only the genes with perfect hit and 99–100% of identity were considered. The virulence factors were identified by VirulenceFinder (https://cge.cbs.dtu.dk/services/VirulenceFinder/), using as criteria of selection 99–100% of identity [91]. The presence of plasmids in the strains under study was determined “in silico” by PlasmidFinder platform (https://cge.cbs.dtu.dk/services/PlasmidFinder/) [92]. The presence of prophages within both bacterial chromosomes was determined by PHAST (http://phast.wishartlab.com/) [39], and PHASTER tools (http://phaster.ca/) [39, 93]. As it is well explained on the website, this server is an update of PHAST, also used for the identification and annotation of prophage sequences present in both the bacterial genome and plasmids. However, both differs in that the PHASTER server being faster since more than 120 CPUs were added to the computational cluster; is more efficient because it has a series of automated algorithms that reduce the size and redundancy of databases; and it is more visually attractive and easier to use, because it has a new graphical genome browser and better tools for visualizing genes and genomes whit a robust and easy-to-use graphical interface. Finally, the search for CRISPR systems was carried out with the CRISPRfinder platform (http://crispr.upsud.fr/Server/CRISPRfinder.php) [40]; while the presence of CRISPR-Cas systems in the genomes were analyzed using the server CRISPR-Cas Meta (https://crisprcas.i2bc.paris-saclay.fr/CrisprCasMeta/Index) [94]

### Antimicrobial susceptibility and plasmid profiles

Antimicrobial susceptibility tests were performed using Müller–Hinton agar (Oxoid, Hampshire, UK) by disk diffusion method. Resistance and sensitivity were interpreted following the standards criteria of the Clinical & Laboratory Standards Institute (CLSI) [95]. The following antimicrobial agents were used: chloramphenicol (30 µg), ampicillin (10 µg), trimethoprim sulfamethoxazole (1.25/23.75 µg), fosfomycin (200 µg), tetracycline (10 µg), kanamycin (30 µg), streptomycin (10 µg), vancomycin (30 µg), nalidixic acid (30 µg), imipenem (10 µg) and gentamycin (10 µg).

The plasmid extraction was performed by the alkaline lysis method as described Kado and Liu (1981) [38]. The plasmids were analyzed by electrophoresis on 0.7% agarose gels, stained with ethidium bromide and UV light exposition. Plasmid molecular size estimation was conducted by calibration curves using the λDNA-HindIII molecular marker (Promega).

### Proteomic analysis

An overnight culture was used to inoculate 50 mL of M9 medium. The CI133 and CI172 strains were grown at 37 °C under static condition until stationary phase. These bacterial cultures were carried out by triplicate. Cells were harvested by centrifugation and resuspended in 5 mL of purification buffer (300 mM NaCl, 30 mM NaH2PO4, pH 8.3) and then were disrupted with a French press. Protein identification was performed by IQUIBICEN/UBA-CONICET, University of Buenos Aires (Buenos Aires, Argentina) through Label-free quantification using Orbitrap. Proteome Discoverer 2.1 and Perseus 1.5.8.5 software were used to analyze the spectrums obtained. Proteins were considered significantly upregulated if they were 2- or more fold change induced for a *p*-value < 0.05. The interactions between two sets of proteins were determined, and the networks were constructed using the STRING v11.5 bioinformatics tool (https://string-db.org/) [96] and taking in account all the types of interactions with a moderate confidence level (0.4).

## Supplementary Information

**Additional file 1: Figure S1.** Phylogenetic analysis of 15 molecular markers performed with the UPGMA method. The analysis involved 25 strains including CI133 and CI172, highlighted in red and blue, respectively. The bootstrap consensus tree inferred from 1000 replicates is taken to represent the evolutionary history of the analyzed strains. The tree is enrooted in *Salmonella enterica* Typhimurium 14028s as the out-group (shown in green). It is shown also the great geographic areas where the strains were isolated and the year of collection in the outermost circle. The tree was performed with MEGA X.

**Additional file 2: Table S1.** Strain specific genes of Shigella flexneri CI133 and CI172 strains.

**Additional file 3: Table S2.** Pangenome of the 82 Shigella flexneri strains studied.

**Additional file 4: Table S3.** Prophage regions identified into the CI133 and CI172 genome.

**Additional file 5: Table S4.** Phage elements identified into intact prophage region of the CI133 genome, by PHASTER software.

**Additional file 6: Figure 3.** C) DNA plasmidic profile. The original picture (left) of this figure was slightly modified for a better visualization of the plasmid bands obtained. These modification include the conversion to its negative mode (inversion of the color of the bands with respect to the background), so that such bands are shown in black on a light background. On the other hand, the last lane of the original picture of the gel was eliminated (broken lines are framed in a white box), since the sample seeded in this lane was not essential to demonstrate our results. The modified figure presented in the manuscript was added to this file for comparison (right panel).

## Data Availability

The Whole Genome Shotgun project of *Shigella flexneri* 2 CI133 strain has been deposited at DDBJ/ENA/GenBank under the accession JAGDQG000000000, the Bioproject accession number is PRJNA498014 and the Biosample accession number is SAMN10275217. While the Whole Genome Shotgun project of *Shigella flexneri* 2 CI172 strain has been deposited at DDBJ/ENA/GenBank under the accession JAGDQF000000000, the Bioproject accession number is PRJNA498020 and BioSample accession number is SAMN10275218.
