# Supplementary material for: Genomic and proteomic characterization of two strains of Shigella flexneri 2 isolated from infants’ stool samples in Argentina
Source: BMC Genomics. 2022 Jul 8;23:495. doi: 10.1186/s12864-022-08711-5 (PMC9264714; doi:10.1186/s12864-022-08711-5)
Supplement: Supplementary file 1 — Additional file 1: Figure S1. Phylogenetic analysis of 15 molecular markers performed with the UPGMA method. The analysis involved 25 strains including CI133 and CI172, highlighted in red and blue, respectively. The bootstrap consensus tree inferred from 1000 replicates is taken to represent the evolutionary history of the analyzed strains. The tree is enrooted in Salmonella enterica Typhimurium 14028s as the out-group (shown in green). It is shown also the great geographic areas where the strains were isolated and the year of collection in the outermost circle. The tree was performed with MEGA X. [file 12864_2022_8711_MOESM1_ESM.ppt]

## Slide 1
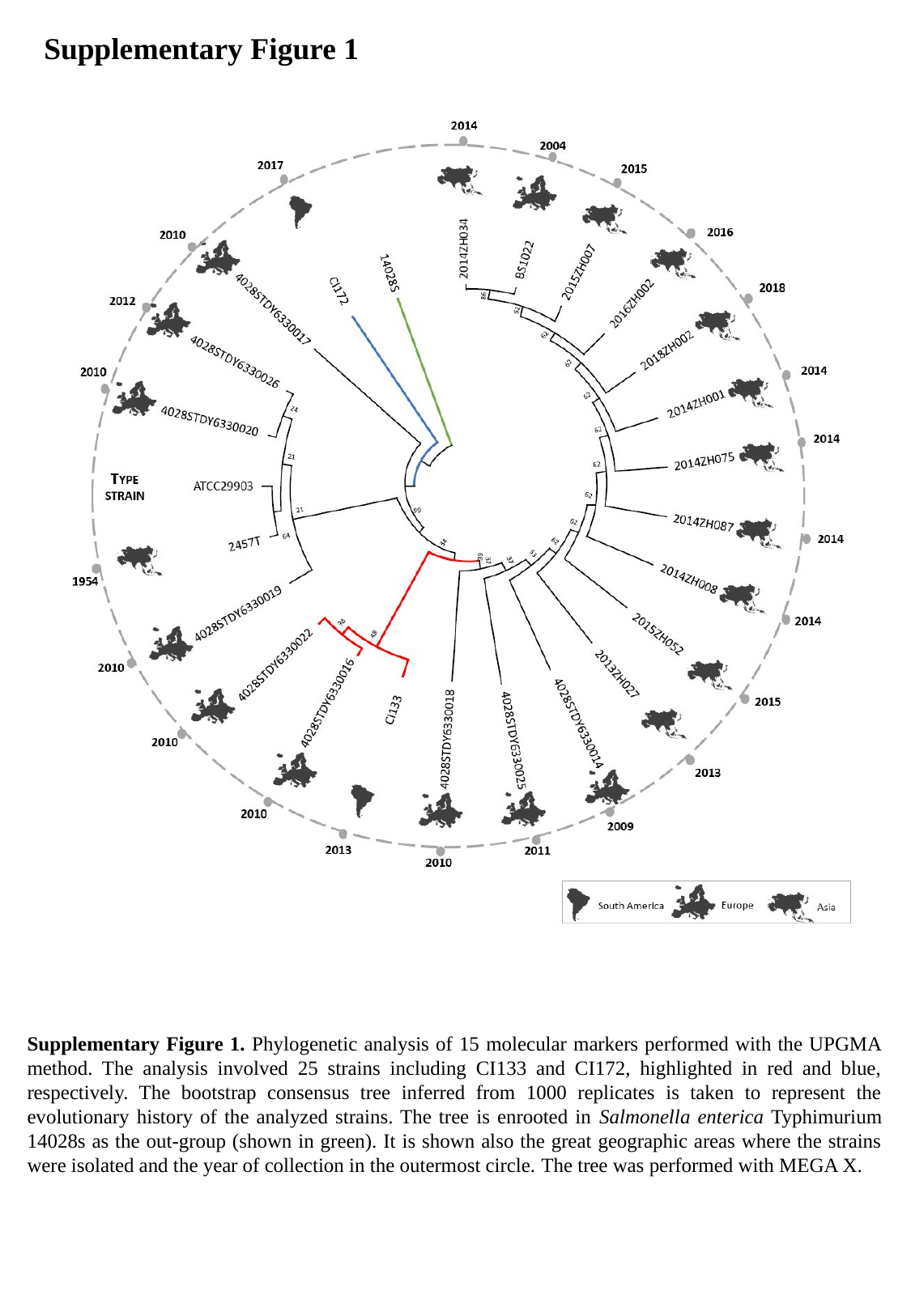

Supplementary Figure 1
Supplementary Figure 1. Phylogenetic analysis of 15 molecular markers performed with the UPGMA method. The analysis involved 25 strains including CI133 and CI172, highlighted in red and blue, respectively. The bootstrap consensus tree inferred from 1000 replicates is taken to represent the evolutionary history of the analyzed strains. The tree is enrooted in Salmonella enterica Typhimurium 14028s as the out-group (shown in green). It is shown also the great geographic areas where the strains were isolated and the year of collection in the outermost circle. The tree was performed with MEGA X.
