# Supplementary material for: Genomic and proteomic characterization of two strains of Shigella flexneri 2 isolated from infants’ stool samples in Argentina
Source: BMC Genomics. 2022 Jul 8;23:495. doi: 10.1186/s12864-022-08711-5 (PMC9264714; doi:10.1186/s12864-022-08711-5)
Supplement: Supplementary file 6 — Additional file 6: Figure 3. C) DNA plasmidic profile. The original picture (left) of this figure was slightly modified for a better visualization of the plasmid bands obtained. These modification include the conversion to its negative mode (inversion of the color of the bands with respect to the background), so that such bands are shown in black on a light background. On the other hand, the last lane of the original picture of the gel was eliminated (broken lines are framed in a white box), since the sample seeded in this lane was not essential to demonstrate our results. The modified figure presented in the manuscript was added to this file for comparison (right panel). [file 12864_2022_8711_MOESM6_ESM.pdf]

Original picture

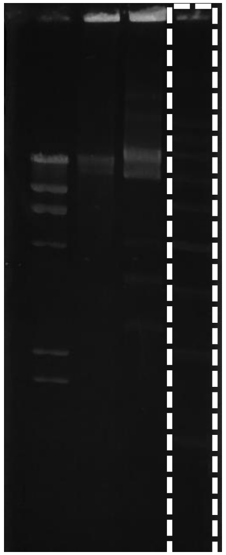

Final picture Fig. 3C

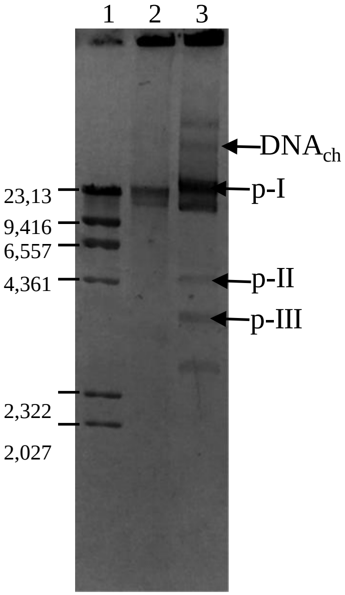

Figure 3 C) DNA plasmidic profile.

The **original picture** (left) of this figure was slightly modified for a better visualization of the plasmid bands obtained. These modification include the conversion to its negative mode (inversion of the color of the bands with respect to the background), so that such bands are shown in black on a light background. On the other hand, the last lane of the original picture of the gel was eliminated (broken lines are framed in a white box), since the sample seeded in this lane was not essential to demonstrate our results. The modified figure presented in the manuscript was added to this file for comparison (right panel).
